# Empagliflozin attenuates AGE-BSA/high-glucose-induced inflammation in C2C12 myotubes associated with suppression of the RAGE/NF-κB pathway

**DOI:** 10.3389/fendo.2026.1903580

**Published:** 2026-08-18

**Authors:** Xiaoying Zhang, Yu Mao, Jiaxing Wu, Xinyu Deng, Huanhuan Wang, Zhiqiang Kang

**Affiliations:** Department of Endocrinology, Zhengzhou Central Hospital Affiliated to Zhengzhou University, Zhengzhou, Henan, China

**Keywords:** AGE-RAGE signaling, C2C12 myotubes, diabetes-related skeletal muscle injury, empagliflozin, inflammation, NF-κB

## Abstract

**Introduction:**

Empagliflozin is an established sodium-glucose cotransporter 2 inhibitor with reported anti-inflammatory activity, but its effects on advanced glycation end product/high-glucose-induced inflammatory injury in skeletal muscle cells remain unclear.

**Methods:**

A GEO-derived transcriptomic dataset from gastrocnemius muscle of type 2 diabetes mellitus rats was analyzed to screen pathways associated with diabetes-related skeletal muscle injury, and molecular docking was performed to explore the possible structural compatibility between empagliflozin and RAGE. Differentiated C2C12 myotubes were exposed to AGE-BSA under high-glucose conditions and treated with empagliflozin. Cell viability was assessed using CCK-8, and RAGE, NF-κB, IL-6, and TNF-α were evaluated by qRT-PCR, Western blotting, immunofluorescence, and ELISA.

**Results:**

Bioinformatic enrichment highlighted AGE-RAGE signaling and inflammation-related NF-κB pathways in diabetic skeletal muscle. Molecular docking provided a computational structural hypothesis suggesting possible compatibility between empagliflozin and human RAGE involving Asp160. In AGE-BSA/high-glucose-stimulated C2C12 myotubes, empagliflozin improved cell viability and was accompanied by reduced RAGE expression, lower NF-κB phosphorylation and immunofluorescence-based nuclear accumulation, and lower IL-6 and TNF-α expression and secretion.

**Discussion:**

Empagliflozin attenuates AGE-BSA/high-glucose-induced inflammatory injury in C2C12 myotubes, and this effect is associated with reduced activation of RAGE/NF-κB-related inflammatory signaling. Further gain- or loss-of-function experiments and validation in diabetic animal models are required to determine whether this pathway is causally involved and whether the observed cell-based effect can be translated into an in vivo setting.

## Introduction

Diabetes mellitus adversely affects skeletal muscle, leading to loss of muscle mass, deterioration of muscle quality, and reduced contractile performance. Persistent hyperglycemia and insulin resistance reshape skeletal muscle metabolism by enhancing inflammatory stress, oxidative injury, and dysregulated protein turnover, thereby participating in diabetes-related muscle damage (1–4).

Advanced glycation end products (AGEs) represent a major link between hyperglycemia and tissue injury. When AGEs engage their receptor RAGE, downstream inflammatory networks can be initiated, among which NF-κB is particularly important. Subsequent induction of mediators such as IL-6 and TNF-α may intensify the inflammatory milieu in skeletal muscle and contribute to diabetic muscle dysfunction. Recent reviews and clinical studies further support the relevance of AGE accumulation, RAGE activation, and musculoskeletal AGE burden to sarcopenia, dynapenia, and diabetes-related muscle impairment (1, 2, 5–15).

Sodium-glucose cotransporter 2 (SGLT2) inhibitors are established glucose-lowering agents, and experimental studies also indicate anti-inflammatory and cell-protective activities. Empagliflozin, one of the commonly used SGLT2 inhibitors, has been associated with suppression of inflammatory mediators and regulation of stress-related signaling. Clinical and translational studies suggest that SGLT2 inhibitors may alter body composition, fat mass, skeletal muscle mass, or muscle metabolic and inflammatory profiles, but their net influence on muscle health remains incompletely resolved. Recent studies have further suggested that empagliflozin may exert muscle-related metabolic effects beyond renal glucose lowering. In skeletal muscle cells, empagliflozin has been reported to promote fatty acid oxidation and alter cellular energy metabolism. In aging skeletal muscle, empagliflozin has been associated with reduced muscle fibrosis through AMPKα-related signaling. In cardiac muscle disease models, empagliflozin has also been reported to improve myocardial metabolism, mitochondrial function, ketone body metabolism, oxidative stress, and ischemia/reperfusion-related cardiac dysfunction (36–39). These findings suggest that the potential effects of empagliflozin on diabetes-related muscle injury may involve both inflammatory and metabolic mechanisms. Nevertheless, the influence of empagliflozin on AGE-RAGE/NF-κB-associated inflammation in skeletal muscle cells under diabetic-like injury conditions has not been fully defined (16–35).

Empagliflozin was selected in the present study because it is one of the most extensively investigated SGLT2 inhibitors and has well-established clinical relevance in patients with type 2 diabetes. In addition to its glucose-lowering effect, previous studies have reported anti-inflammatory and tissue-protective properties of empagliflozin, including modulation of NF-κB-related inflammatory signaling. Because the present study focused on AGE-BSA/high-glucose-induced inflammatory injury and RAGE/NF-κB-related responses in skeletal muscle cells, empagliflozin was considered a reasonable representative SGLT2 inhibitor for this preliminary *in vitro* investigation.

Here, GEO-based pathway screening, molecular docking, and an AGE-BSA/high-glucose C2C12 myotube model were integrated to evaluate whether empagliflozin attenuates inflammatory injury and whether this response is associated with changes in RAGE/NF-κB signaling. This study was designed as a cell-level inflammatory injury investigation rather than a causal pathway-validation study or a full *in vivo* diabetic sarcopenia model.

## Materials and methods

### Transcriptomic data acquisition and preprocessing

RNA-sequencing data from gastrocnemius muscle of type 2 diabetes mellitus (T2DM) rats were obtained from the GEO database under accession GSE268372. The dataset comprised six samples, including three normal controls and three T2DM model rats, generated on the Illumina NovaSeq 6000 platform (GPL25947) for Rattus norvegicus. After removal of adaptor-contaminated and low-quality reads, clean reads were mapped to the rat reference genome (Rnor_6.0) with HISAT2. Gene counts were summarized by featureCounts and converted to FPKM values for normalization. Downstream analyses were conducted in R version 4.1.2.

### Differential gene expression analysis

Differentially expressed genes were identified by comparing the T2DM and Control groups in R. Genes were considered DEGs when |log2FC| exceeded 1 and P was less than 0.05. The global DEG distribution was displayed in a volcano plot generated with ggplot2; upregulated, downregulated, and nonsignificant genes were shown in different colors. Representative DEGs involved in extracellular matrix remodeling, inflammatory processes, and metabolic regulation were further displayed in a pheatmap heatmap after row-wise scaling of normalized expression values and Euclidean-distance clustering.

### Functional enrichment analysis

GO and KEGG enrichment analyses were carried out for the DEG set using clusterProfiler (version 4.2.2) in R. GO enrichment covered the biological process, cellular component, and molecular function categories, whereas KEGG analysis was used to identify disease- and signaling-related pathways that might participate in diabetic skeletal muscle injury. Terms or pathways with adjusted P values below 0.05 were regarded as significant. The top 10 GO terms were displayed as horizontal bar plots, and the top 10 enriched KEGG pathways were shown as a bubble chart, with point size representing gene count and color indicating enrichment significance.

### Molecular docking simulation

The crystal structure of human RAGE protein, encoded by the *AGER* gene, was downloaded from the RCSB Protein Data Bank (PDB ID: 4LP5; UniProt accession Q15109), and the empagliflozin structure was retrieved from PubChem (CID: 9887712). Prior to docking, PyMOL 2.5.0 was used to delete water molecules and co-crystallized ligands from the RAGE structure. AutoDockTools 1.5.7 was then used to add hydrogen atoms and Gasteiger charges to both protein and ligand, and rotatable bonds were assigned for empagliflozin.

AutoDock Vina 1.2.5 was used to model the possible docking pose of empagliflozin within the human RAGE structure. The search box was placed around Asp160, with dimensions of 40 × 40 × 40 Å and a spacing of 1.0 Å; exhaustiveness was set to 8. The pose with the lowest predicted binding energy was selected for graphical presentation, and PyMOL 2.5.0 was used to evaluate hydrogen bonds and distances. Because a human RAGE structure was used for the docking simulation but the cell experiments were conducted in mouse C2C12 myotubes, the docking findings were treated as a structural hypothesis rather than direct proof of target engagement.

### Cell culture and model construction

Mouse C2C12 myoblasts (Cat. No. SCC20027; Zishan Biotechnology Company, Wuhan, China) were cultured in high-glucose DMEM containing 25 mmol/L D-glucose and supplemented with 10% fetal bovine serum and 1% penicillin-streptomycin. The cell lines present in this study were obtained from Zishan Biotechnology Company, Wuhan, China.

To induce myotube formation, adherent C2C12 myoblasts that had not yet fused were switched to DMEM supplemented with 2% horse serum and maintained for 5 days. During the subsequent experimental period, cells in the blank control group received 5.55 mmol/L glucose, whereas the model and empagliflozin-treated groups were exposed to 30 mmol/L glucose. AGE-BSA (Cat. No. 22968; Cayman Chemical Company, Ann Arbor, MI, USA) was added at 200 μg/mL during the final 48h. To evaluate whether BSA itself affected RAGE expression, an additional native non-glycated BSA control experiment was performed. Differentiated C2C12 myotubes were treated with native non-glycated BSA (Servicebio, Wuhan, China; Cat. No. GC305006) at 200 μg/mL for 48h, which matched the concentration and duration of AGE-BSA treatment. RAGE mRNA expression was then assessed by qRT-PCR using the same protocol and primers described below. Empagliflozin (HY-15409; MedChemExpress, Monmouth Junction, NJ, USA; CAS No. 864070-44-0) was dissolved in dimethyl sulfoxide (DMSO) to prepare a stock solution and then diluted with culture medium to the indicated working concentrations. The final DMSO concentration was kept identical among empagliflozin-treated and solvent control groups and did not exceed 0.1% (v/v). For drug screening, AGE-BSA/high-glucose-treated C2C12 myotubes were exposed to empagliflozin at 1, 10, 20, 30, or 50 μM for 48h. For subsequent experiments, empagliflozin was applied at 10, 20, or 50 μM concurrently with AGE-BSA/high-glucose stimulation for 48h.

### Cell viability assay

Cell viability was assessed using a CCK-8 Plus assay kit (Cat. No. G1613-1ML; Servicebio, Wuhan, China) according to the manufacturer’s instructions. Briefly, C2C12 myotubes were cultured in 96-well plates and subjected to the indicated treatments. At the end of treatment, 10 μL of CCK-8 Plus reagent was added to each well, with care taken to avoid bubble formation because bubbles may interfere with absorbance measurements. The plates were incubated at 37°C for 0.5h in a cell culture incubator. The optical density was then measured at 450 nm using a microplate reader. Background absorbance from wells containing medium and reagent but no cells was subtracted, and cell viability was calculated relative to the blank control group.

### Western blotting

For Western blotting, C2C12 myotubes were harvested in RIPA lysis buffer (Cat. No. G2033; Sivell Bio, Wuhan, China) containing PMSF at 1:100 and phosphatase inhibitor at 1:50. Protein samples of equal quantity (10 μg) were resolved by 10% SDS-PAGE and electrotransferred to PVDF membranes (Cat. No. G6047; Sivell Bio, Wuhan, China) under constant voltage at 100V for 80min. Membranes were blocked with milk solution for 2h at room temperature and then incubated overnight at 4°C with primary antibodies. After washing, membranes were reacted for 1h at room temperature with HRP-conjugated goat anti-rabbit or goat anti-mouse IgG secondary antibodies (1:5000; Proteintech Group, Wuhan, China). Signals were developed with enhanced chemiluminescence reagent (Cat. No. SQ101; Epizyme, China), and band density was analyzed with ImageJ 1.54s14 (National Institutes of Health, Bethesda, MD, USA). Western blot images shown in the main figures were cropped only for presentation. No individual band or lane was selectively enhanced, obscured, moved, removed, or added. When brightness or contrast adjustment was necessary, it was applied uniformly to the entire image. Original full-length blot scans were retained and prepared for submission as **Supplementary Material**.

The following primary antibodies were used: mouse anti-GAPDH (Cat. No. HRP-60004; 1:10000; Proteintech Group, Wuhan, China), rabbit anti-RAGE (Cat. No. 16346-1-AP; 1:5000; Proteintech Group, Wuhan, China), rabbit anti-NF-κB (Cat. No. 80979-1-RR; 1:10000; Proteintech Group, Wuhan, China), and rabbit anti-phospho-NF-κB (Cat. No. TP56372S; 1:1000; ABmart).

### Real-time quantitative polymerase chain reaction

Total RNA was isolated from C2C12 myotubes with VeZol Reagent (Cat. No. R411-01; Vazyme Biotech Co., Ltd., Nanjing, China). RNA quantity and purity were assessed by the 260/280 nm absorbance ratio using a NanoDrop spectrophotometer (Thermo Scientific, USA). For each sample, 1 μg RNA was converted into cDNA using a reverse transcription kit (Cat. No. MR101-01; Vazyme Biotech Co., Ltd., Nanjing, China).

Real-time PCR was performed using a QuantStudio 6 Pro Real-Time PCR System (Applied Biosystems, Thermo Fisher Scientific, USA). The cycling protocol included 30 s at 95°C for initial denaturation, followed by 40 cycles of 95°C for 5 s and 60°C for 30 s. *Gapdh* was used as the internal reference gene, and relative mRNA abundance was calculated using the 2^(-ΔΔCt) approach. Each qRT-PCR reaction was performed in technical triplicate, and the mean Ct value was used for relative expression analysis. Primer information is provided in **Table 1**.

**Table 1:** qRT-PCR primer sequences.

Gene	Forward primer (5′-3′)	Reverse primer (5′-3′)
*Ager*	CTTGCTCTATGGGGAGCTGTA	GGAGGATTTGAGCCACGCT
*Nfkb1*	ATGGCAGACGATGATCCCTAC	TGTTGACAGTGGTATTTCTGGTG
*Il6*	CCAAGAGGTGAGTGCTTCCC	CTGTTGTTCAGACTCTCTCCCT
*Tnf*	GACGTGGAACTGGCAGAAGAG	TTGGTGGTTTGTGAGTGTGAG
*Gapdh*	AGGTCGGTGTGAACGGATTTG	TGTAGACCATGTAGTTGAGGTCA

### Immunofluorescence

For immunofluorescence staining, C2C12 myotubes grown on sterile coverslips were fixed with 4% paraformaldehyde and permeabilized with 0.1% Triton X-100/PBS. Nonspecific binding was blocked using PBS containing 1% glycine and 3% BSA, after which cells were incubated overnight at 4°C in a humidified chamber with rabbit anti-NF-κB antibody diluted at 1:150 in PBS containing 3% BSA. On the following day, cells were incubated in the dark with an Alexa Fluor 594-conjugated secondary antibody (Cat. No. GB28303; Servicebio, Wuhan, China) diluted at 1:400 in PBS containing 3% BSA. Nuclei were stained with DAPI, and the coverslips were mounted in fluorescent mounting medium composed of 80% glycerol and 20% PBS. Images were captured with a Zeiss LSM 900 laser scanning confocal microscope (Carl Zeiss AG, Oberkochen, Germany).

For quantitative analysis, images were acquired using identical microscope settings within the same experiment. NF-κB fluorescence intensity was quantified using ImageJ. Nuclear regions were defined according to DAPI staining, and cytoplasmic regions were selected from the corresponding cell area excluding the nucleus. The nuclear-to-cytoplasmic NF-κB fluorescence intensity ratio was calculated for each image and used to assess immunofluorescence-based NF-κB nuclear accumulation. Multiple randomly selected fields were analyzed for each biological replicate, and the average value was used for statistical analysis.

### Enzyme-linked immunosorbent assay

Culture medium was collected after treatment and centrifuged at 1000 × g for 10min to remove cell debris. The concentrations of IL-6, TNF-α, and MCP-1 in the supernatants were measured using commercial ELISA kits from Jianglai Biological Technology (China) according to the manufacturer’s instructions. The following kits were used: mouse TNF-α ELISA kit (Cat. No. JL10484; detection range, 15.62–1000 pg/mL; sensitivity, 7.26 pg/mL), mouse IL-6 ELISA kit (Cat. No. JL20268; detection range, 7.81–500 pg/mL; sensitivity, 2.63 pg/mL), and mouse MCP-1/CCL2 ELISA kit (Cat. No. JL20304; detection range, 31.25–2000 pg/mL; sensitivity, 11.76 pg/mL). Standards and samples were added to ELISA plates according to the kit protocols, followed by sequential incubation with detection reagents, substrate solution, and stop solution. Absorbance was measured at 450 nm using a microplate reader, and cytokine concentrations were calculated from standard curves. Technical wells were averaged before statistical analysis. MCP-1 was measured under the same experimental conditions as an additional inflammatory chemokine marker and is presented in **Supplementary Figure 3**.

### Statistical analysis

Data analysis was performed in GraphPad Prism 10.0 (GraphPad Software Inc., La Jolla, CA, USA). Results are expressed as mean ± SD. Each experiment was repeated independently at least three times. Unless otherwise specified, n denotes independent biological replicates, with each replicate representing an independently prepared and treated cell culture sample. Technical measurements or technical wells, when performed, were not treated as independent biological replicates for statistical analysis. Normality and variance homogeneity were checked using the Shapiro-Wilk and Levene tests before intergroup comparisons. When these assumptions were met, multiple-group comparisons were analyzed by one-way ANOVA followed by appropriate *post hoc* multiple-comparison tests. For cell-based intervention experiments, the AGE-BSA/high-glucose model group was compared with the blank control group, and empagliflozin-treated groups were compared with the AGE-BSA/high-glucose model group. If the assumptions were not satisfied, the Kruskal-Wallis test followed by appropriate *post hoc* comparisons was applied. Statistical significance was defined as P < 0.05.

## Results

### Bioinformatic screening of AGE-RAGE/NF-κB-related pathways and docking-based structural hypothesis for empagliflozin-RAGE compatibility

To explore pathways involved in diabetes-related skeletal muscle injury, transcriptomic profiles from gastrocnemius muscle of control and T2DM rats were analyzed using the GEO dataset.

**Figure 1:**
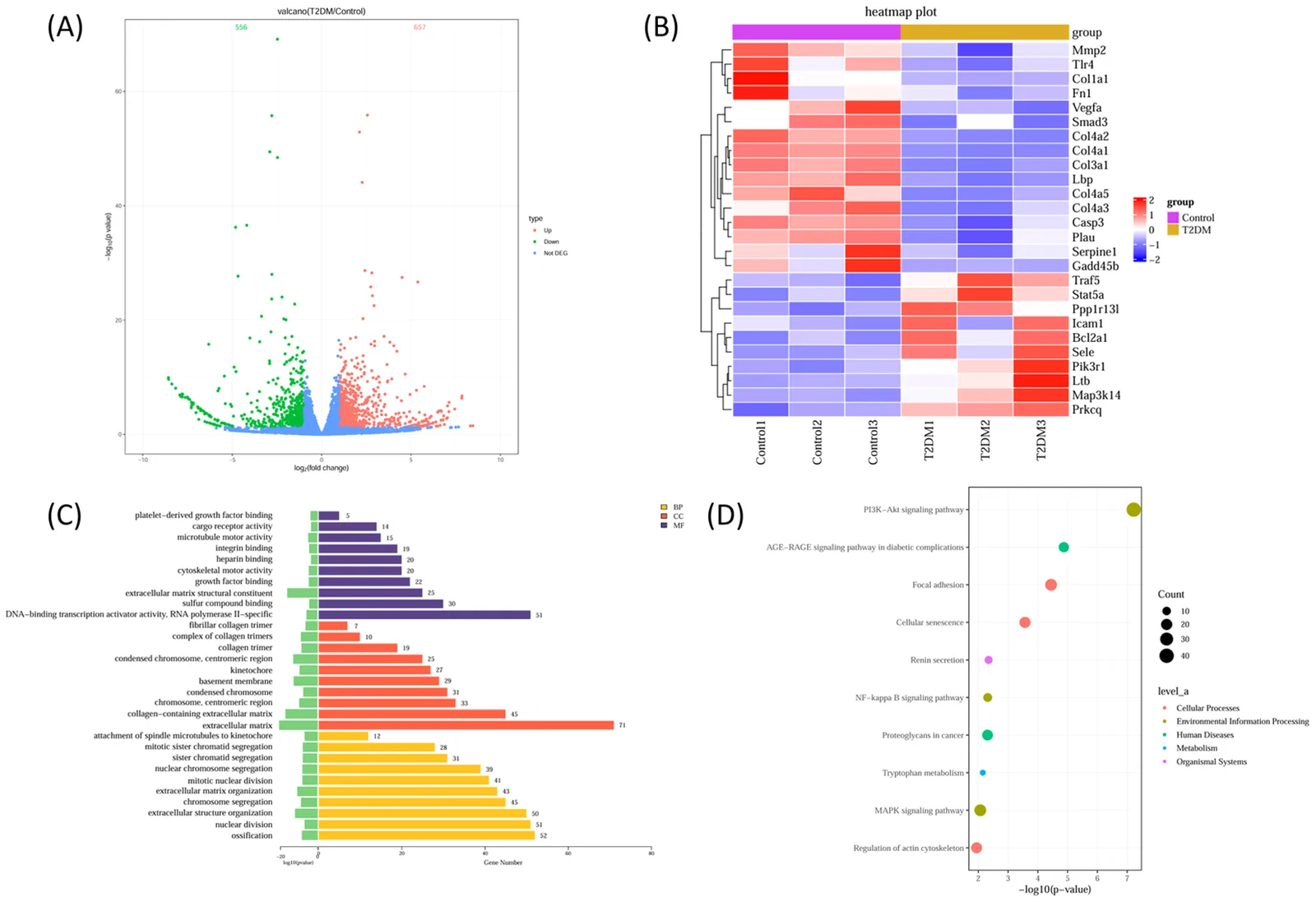
Differential expression and functional enrichment results for diabetic skeletal muscle compared with control tissue. **(A)** Volcano plot showing DEGs screened using |log2FC| > 1 and P < 0.05. Red, green, and blue points denote significantly upregulated genes, significantly downregulated genes, and genes without significant differential expression, respectively. The numbers of increased and decreased genes are displayed in the plot. **(B)** Heatmap displaying selected DEGs in Control and T2DM samples. The color gradient reflects scaled normalized expression, with red indicating higher and blue indicating lower expression. Columns represent individual samples and rows represent genes. **(C)** GO enrichment of DEGs. The 10 leading terms are shown across biological process, cellular component, and molecular function categories, with the x-axis indicating the number of genes included in each term. **(D)** KEGG enrichment of DEGs. Dot size corresponds to the number of genes assigned to each pathway, and dot color represents pathway category. The AGE-RAGE signaling pathway in diabetic complications is indicated. DEG, differentially expressed gene; GO, Gene Ontology; KEGG, Kyoto Encyclopedia of Genes and Genomes; AGE-RAGE, advanced glycation end product-receptor for advanced glycation end products.

With |log2FC| > 1 and P < 0.05 as cutoffs, 623 genes showed higher expression and 566 genes showed lower expression in T2DM samples relative to controls (**Figure 1A**). A heatmap of representative genes linked to extracellular matrix remodeling, inflammation, and metabolism revealed clear differences between the two groups (**Figure 1B**).

GO analysis of the leading enriched terms indicated that many DEGs were involved in extracellular matrix organization, collagen-associated processes, and transcriptional regulation (**Figure 1C**).

KEGG analysis identified several pathways with potential relevance to diabetic muscle injury, including AGE-RAGE signaling in diabetic complications, NF-κB signaling, PI3K-Akt signaling, and focal adhesion (**Figure 1D**).

Although PI3K-Akt signaling was also prominently enriched in the KEGG analysis, the present study focused on the AGE-RAGE/NF-κB axis because this pathway was more directly aligned with the AGE-BSA/high-glucose injury model and the inflammatory endpoints measured in the subsequent cell experiments. AGE-BSA is a direct upstream stimulus for RAGE activation, and RAGE activation is closely linked to NF-κB-mediated inflammatory responses. Therefore, AGE-RAGE/NF-κB signaling was selected as the primary pathway for experimental assessment in this study, whereas PI3K-Akt signaling was regarded as another potentially relevant pathway requiring future investigation.

Although PPI-based hub-gene ranking may provide useful information regarding the topological importance of differentially expressed genes, the present study did not establish RAGE as a topological hub within a DEG-based PPI network. Therefore, the selection of RAGE as the molecular docking target was primarily pathway-guided and model-driven rather than network-topology-driven. Specifically, KEGG enrichment analysis highlighted the AGE-RAGE signaling pathway in diabetic skeletal muscle, and the subsequent cellular injury model was established using AGE-BSA under high-glucose conditions. Because RAGE is the canonical receptor mediating AGE-induced inflammatory signaling and is closely linked to downstream NF-κB-related inflammatory responses, RAGE was selected as a biologically relevant receptor for exploratory molecular docking. Accordingly, the docking analysis should be interpreted as a hypothesis-generating structural prediction rather than evidence that RAGE represents the topological core of the DEG network.

Together, these bioinformatic results supported further evaluation of whether empagliflozin treatment is accompanied by changes in AGE-RAGE/NF-κB-associated inflammatory signaling in skeletal muscle cells.

**Figure 2:**
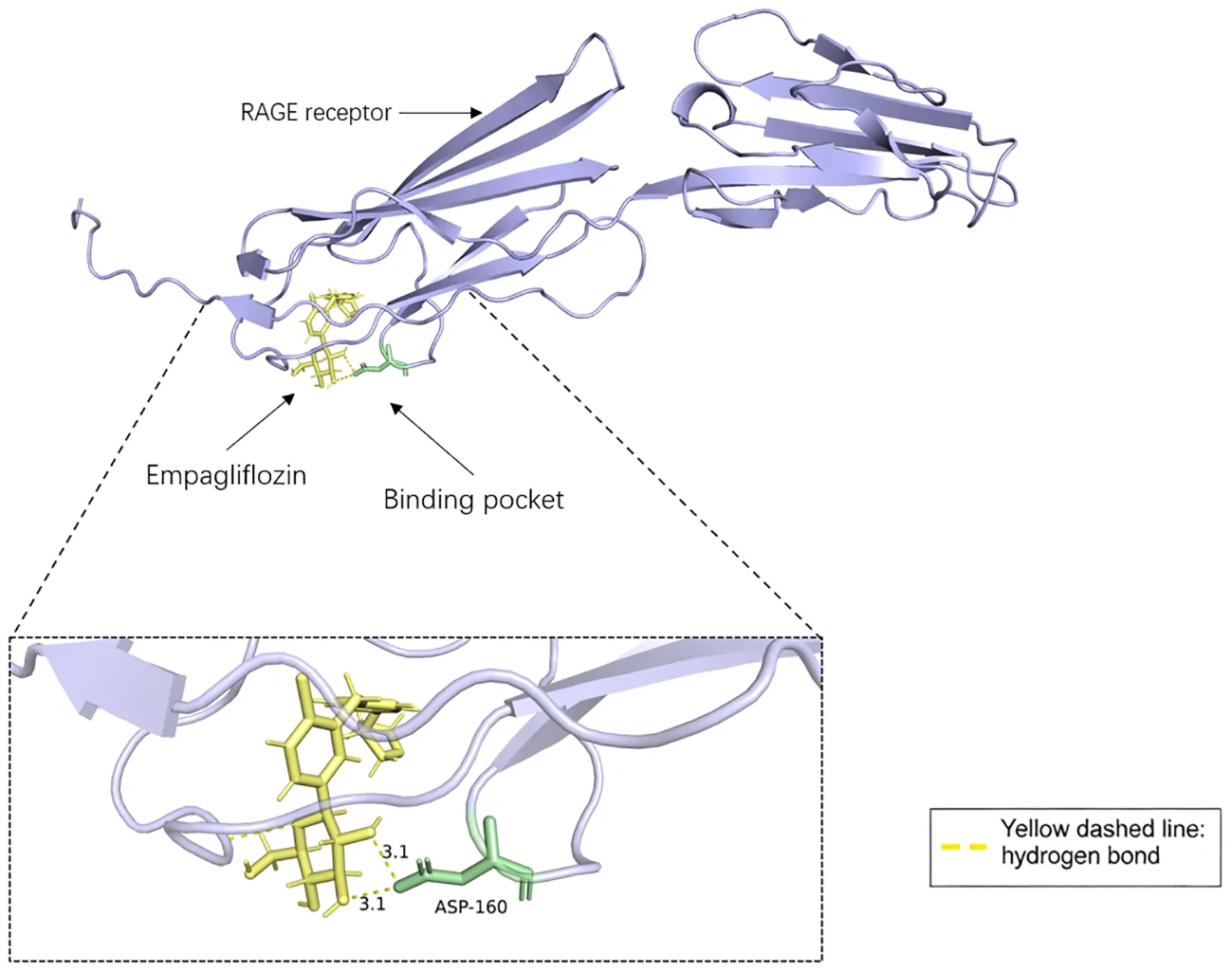
Docking-based structural prediction of potential compatibility between empagliflozin and human RAGE. The ribbon structure represents the extracellular domain of human RAGE, and empagliflozin is displayed as sticks within the predicted pocket. The enlarged panel shows the predicted contact region between empagliflozin and human RAGE. This docking model provides computational structural information only and should not be taken as proof of direct binding, target engagement, or functional regulation of the RAGE/NF-κB pathway. RAGE, receptor for advanced glycation end products; NF-κB, nuclear factor-kappa B.

Docking analysis was subsequently conducted to generate a computational structural hypothesis regarding the potential compatibility between empagliflozin and RAGE. In the optimal predicted pose, empagliflozin was positioned within a pocket of the human RAGE structure and was predicted to form a hydrogen bond with Asp160 at an estimated distance of 3.1 Å (**Figure 2**). This computational result suggests possible structural compatibility between empagliflozin and human RAGE. However, because the docking analysis was based on a human RAGE structure whereas the cell experiments were performed in mouse C2C12 myotubes, and because no direct binding assay was conducted, this result should be interpreted only as a hypothesis-generating prediction rather than experimental confirmation of direct binding or pathway blockade.

### Establishment of the AGE-BSA/high-glucose C2C12 myotube injury model and selection of empagliflozin treatment conditions

**Figure 3:**
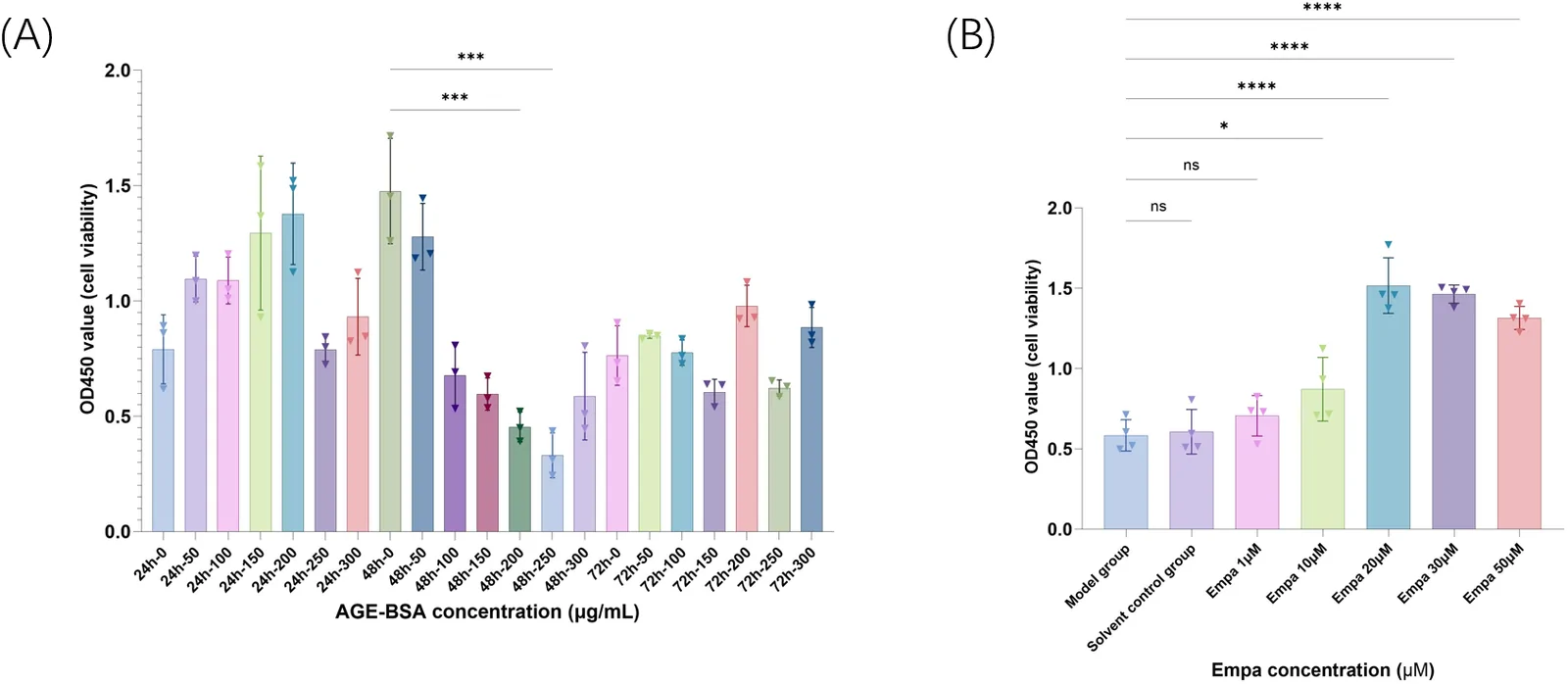
CCK-8 screening of AGE-BSA modeling conditions and empagliflozin treatment doses. **(A)** C2C12 myotubes were exposed to 0-300 μg/mL AGE-BSA for 24, 48, or 72h, after which viability was measured by CCK-8. The 200 μg/mL AGE-BSA and 48h condition was chosen as the standard injury model for later assays. **(B)** Viability of C2C12 myotubes exposed to AGE-BSA/high-glucose and treated with 0-50 μM empagliflozin for 48h. Values are shown as mean ± SD. Statistical comparisons are indicated by connecting lines above the bars. ns, not significant; *P < 0.05; **P < 0.01; ***P < 0.001; ****P < 0.0001. CCK-8, Cell Counting Kit-8; AGE-BSA, advanced glycation end product-bovine serum albumin.

C2C12 myotubes were incubated with AGE-BSA at 0, 50, 100, 150, 200, 250, or 300 μg/mL for 24, 48, or 72h, followed by CCK-8 assessment of cell viability (**Figure 3A**).

AGE-BSA decreased viability in a concentration- and time-dependent manner. Exposure to 200 μg/mL AGE-BSA for 48h produced an approximately 50% decrease in viability while avoiding extensive cell loss; therefore, this condition was used for the following experiments.

### Empagliflozin treatment was associated with improved viability of C2C12 myotubes exposed to AGE-BSA/high-glucose

For drug screening, AGE-BSA/high-glucose-treated myotubes received empagliflozin at 1, 10, 20, 30, or 50 μM for 48h before CCK-8 detection (**Figure 3B**).

The solvent control was comparable to the model group and did not alter cell viability significantly.

Empagliflozin partially improved cell viability under AGE-BSA/high-glucose stress, and a clear protective response was evident from 10 μM upward.

The 20 μM group showed the greatest improvement, whereas 30 and 50 μM remained beneficial but did not further increase viability beyond the 20 μM response.

Accordingly, 20 μM empagliflozin was chosen as the principal concentration for subsequent mechanistic experiments.

### Empagliflozin treatment was associated with reduced RAGE/NF-κB-related gene expression and inflammatory mediators in C2C12 myotubes

qRT-PCR was used to measure *Ager* and cytokine mRNA levels in C2C12 myotubes treated with AGE-BSA/high-glucose in the presence or absence of empagliflozin.

### *Ager* mRNA expression

**Figure 4:**
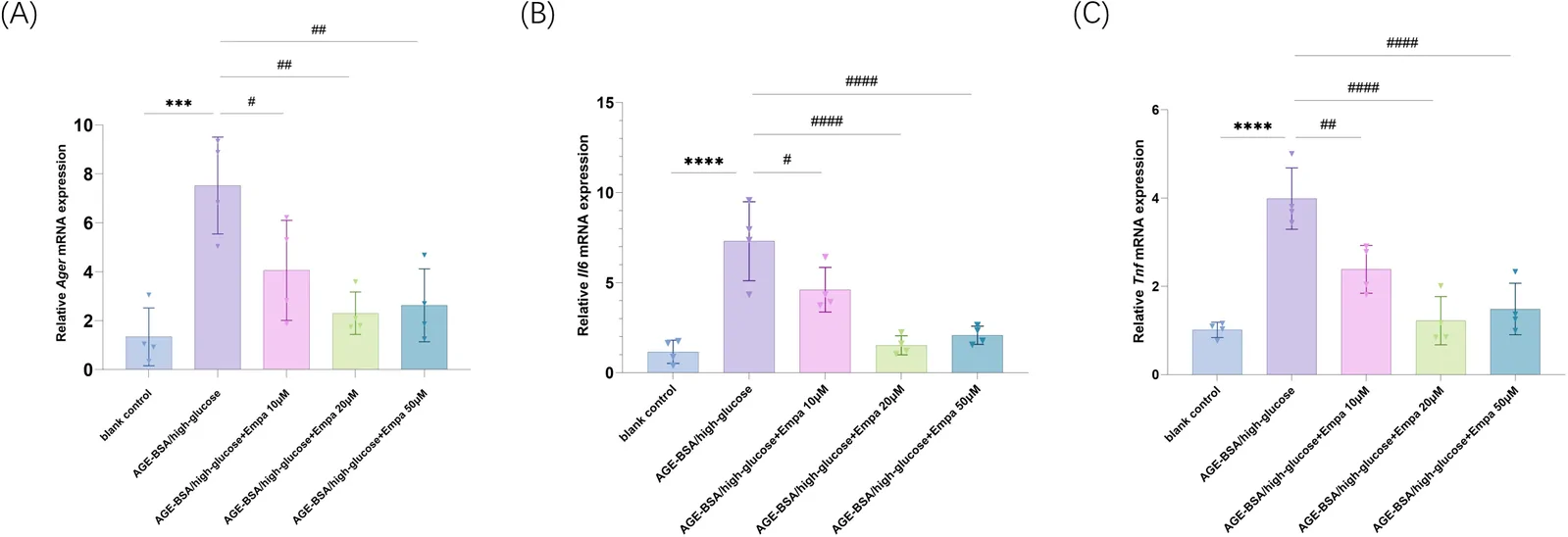
Empagliflozin treatment was associated with reduced *Ager*, *Il6*, and *Tnf* mRNA expression in AGE-BSA/high-glucose-stimulated C2C12 myotubes. **(A–C)** qRT-PCR detection of relative *Ager* **(A)**, *Il6* **(B)**, and *Tnf* **(C)** mRNA abundance. Cells received blank control treatment, AGE-BSA/high-glucose, or AGE-BSA/high-glucose combined with empagliflozin at 10, 20, or 50 μM. Results are presented as mean ± SD (n = 4 independent biological replicates per group). ns, not significant; *P < 0.05, **P < 0.01, ***P < 0.001, ****P < 0.0001 versus the blank control group; ^#^P < 0.05, ^##^P < 0.01, ^###^P < 0.001, ^####^P < 0.0001 versus the AGE-BSA/high-glucose model group. qRT-PCR, quantitative real-time polymerase chain reaction; AGE-BSA, advanced glycation end product-bovine serum albumin. *Ager*, *Il6*, and *Tnf* indicate mouse gene symbols.

Relative to blank controls, the AGE-BSA/high-glucose model showed a significant increase in *Ager* mRNA expression, whereas empagliflozin treatment was associated with lower *Ager* mRNA expression, with the largest decrease observed at 20 μM (**Figure 4A**). In the additional native non-glycated BSA control experiment, native BSA did not significantly alter *Ager* mRNA expression compared with the blank control group (**Supplementary Figure 2**). This result suggests that BSA itself did not markedly induce RAGE expression under the present experimental conditions.

### Expression of inflammatory cytokine genes

*Il6* and *Tnf* mRNA levels were markedly higher in the model group than in blank controls (**Figures 4B, C**).

Empagliflozin treatment was associated with lower *Il6* and *Tnf* mRNA abundance in myotubes subjected to AGE-BSA/high-glucose stimulation.

For *Il6*, significant inhibition was detected at 10, 20, and 50 μM, and the 20 μM group showed the most prominent response (**Figure 4B**).

For *Tnf*, the tested doses of empagliflozin produced significant decreases, especially at 20 and 50 μM (**Figure 4C**).

These data suggest that empagliflozin treatment was associated with lower inflammatory gene expression in this cell-based model of diabetes-related injury. The reduction in cytokine gene expression paralleled the decline in RAGE expression, but these results do not establish a causal requirement for RAGE signaling.

**Figure 5:**
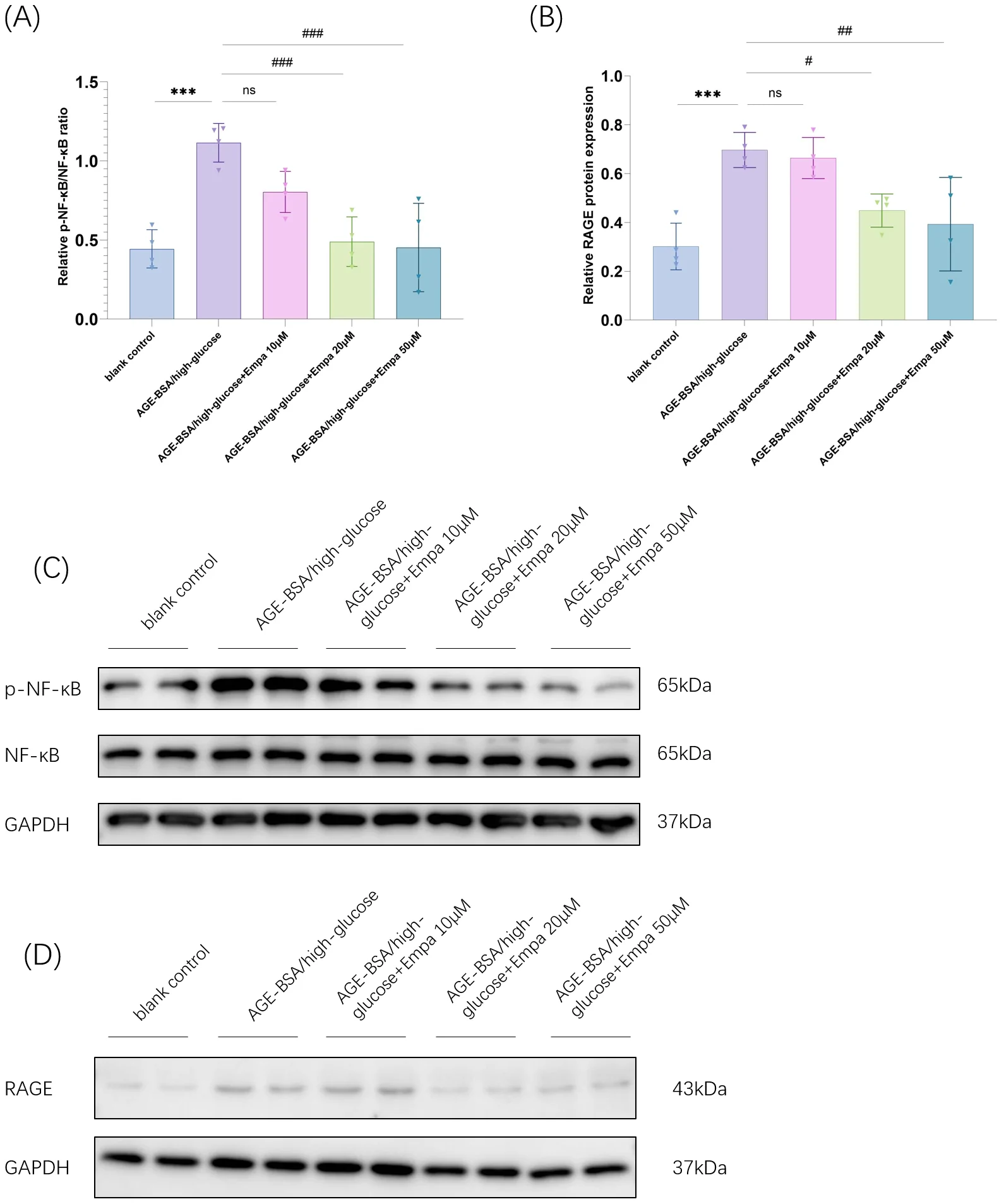
Empagliflozin treatment was associated with reduced RAGE expression and NF-κB phosphorylation in C2C12 myotubes. C2C12 myotubes were assigned to blank control, AGE-BSA/high-glucose model, or AGE-BSA/high-glucose plus empagliflozin (10, 20, or 50 μM) groups. **(A)** Relative NF-κB phosphorylation was expressed as p-NF-κB/NF-κB. **(B)** RAGE protein abundance was normalized to GAPDH. **(C)** Representative Western blot images of p-NF-κB, NF-κB, and GAPDH. **(D)** Representative Western blot images of RAGE and GAPDH. Data are shown as mean ± SD (n = 4 independent biological replicates per group). ns, not significant; *P < 0.05, **P < 0.01, ***P < 0.001, ****P < 0.0001 versus the blank control group; ^#^P < 0.05, ^##^P < 0.01, ^###^P < 0.001, ^####^P < 0.0001 versus the AGE-BSA/high-glucose model group. RAGE, receptor for advanced glycation end products; NF-κB, nuclear factor-kappa B; p-NF-κB/NF-κB, ratio of phosphorylated NF-κB to total NF-κB; GAPDH, glyceraldehyde-3-phosphate dehydrogenase; AGE-BSA, advanced glycation end product-bovine serum albumin.

Western blot analysis further demonstrated that AGE-BSA/high-glucose elevated RAGE, NF-κB, and p-NF-κB protein levels. Empagliflozin treatment was associated with lower levels of these proteins, most obviously at the intermediate and higher doses (**Figure 5**).

### Empagliflozin treatment was associated with reduced NF-κB nuclear accumulation

**Figure 6:**
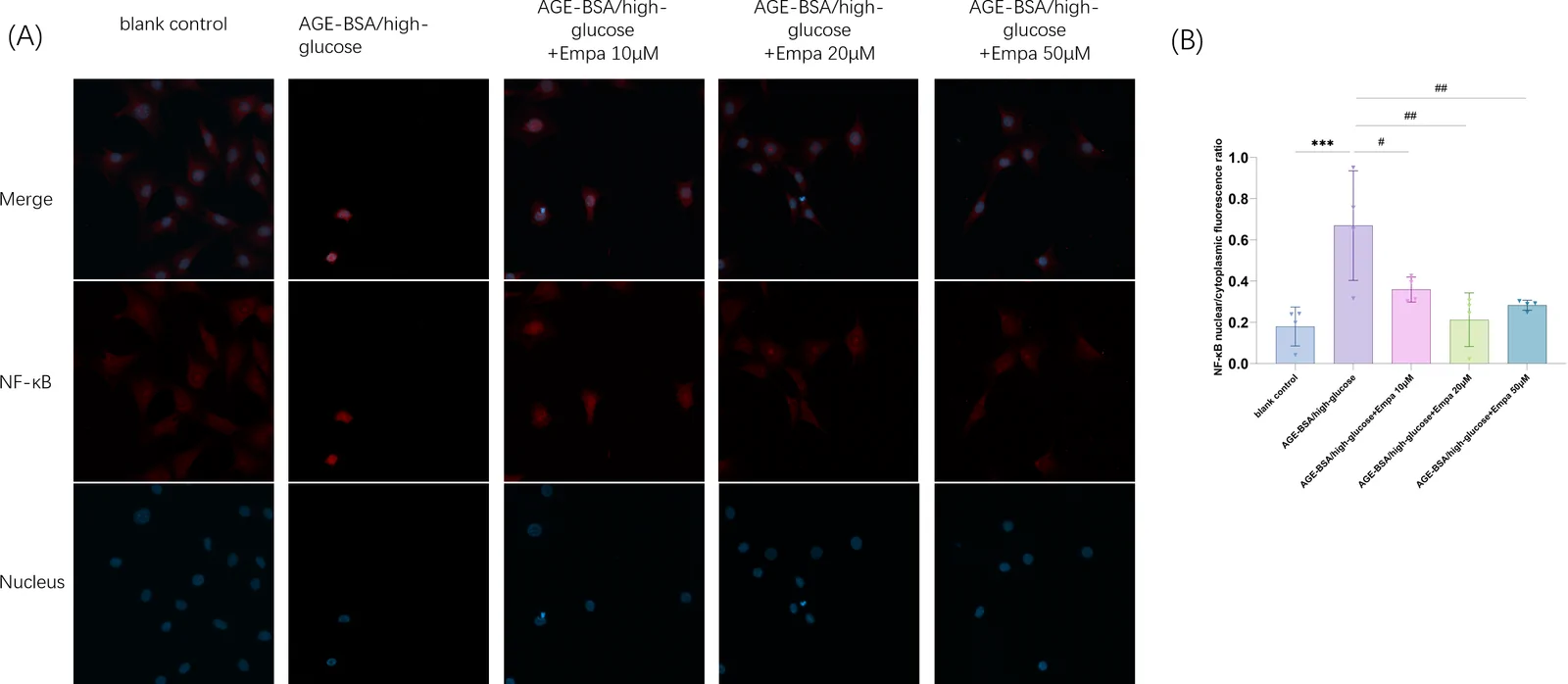
Empagliflozin treatment was associated with reduced NF-κB nuclear accumulation in C2C12 myotubes under AGE-BSA/high-glucose stress. **(A)** Representative immunofluorescence images showing NF-κB distribution in C2C12 myotubes. DAPI was used to stain nuclei. Cells were treated with blank control, AGE-BSA/high-glucose, or AGE-BSA/high-glucose combined with empagliflozin. **(B)** Quantitative analysis of the nuclear-to-cytoplasmic NF-κB fluorescence intensity ratio. AGE-BSA/high-glucose increased immunofluorescence-based NF-κB nuclear accumulation compared with the blank control, whereas empagliflozin treatment was associated with reduced nuclear NF-κB accumulation. Results are expressed as mean ± SD (n = 4 independent biological replicates per group). ns, not significant; *P < 0.05, **P < 0.01, ***P < 0.001, ****P < 0.0001 versus the blank control group; ^#^P < 0.05, ^##^P < 0.01, ^###^P < 0.001, ^####^P < 0.0001 versus the AGE-BSA/high-glucose model group. NF-κB, nuclear factor-kappa B; DAPI, 4′,6-diamidino-2-phenylindole; AGE-BSA, advanced glycation end product-bovine serum albumin.

Empagliflozin treatment was associated with reduced nuclear accumulation of NF-κB in a concentration-dependent pattern (**Figure 6**).

### Empagliflozin treatment was associated with lower IL-6 and TNF-α release

**Figure 7:**
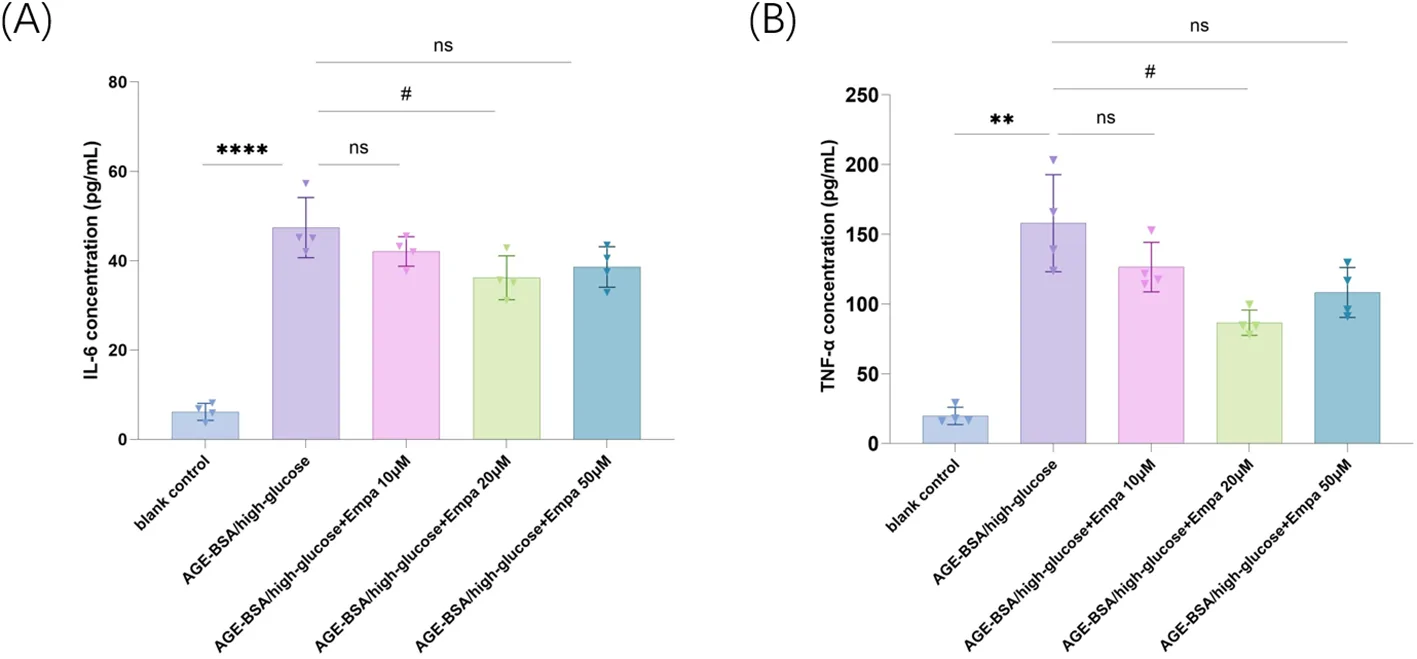
Empagliflozin treatment was associated with lower AGE-BSA/high-glucose-induced secretion of IL-6 and TNF-α in C2C12 myotubes. **(A)** ELISA measurement of IL-6 concentrations in culture supernatants. **(B)** ELISA measurement of TNF-α concentrations in culture supernatants. C2C12 myotubes were treated with blank control, AGE-BSA/high-glucose, or AGE-BSA/high-glucose plus empagliflozin (10, 20, or 50 μM). IL-6 and TNF-α increased after AGE-BSA/high-glucose exposure compared with the blank control, whereas empagliflozin treatment was associated with lower levels of both cytokines. Data are presented as mean ± SD (n = 4 independent biological replicates per group). ns, not significant; *P < 0.05, **P < 0.01, ***P < 0.001, ****P < 0.0001 versus the blank control group; ^#^P < 0.05, ^##^P < 0.01, ^###^P < 0.001, ^####^P < 0.0001 versus the AGE-BSA/high-glucose model group. ELISA, enzyme-linked immunosorbent assay; AGE-BSA, advanced glycation end product-bovine serum albumin; IL-6, interleukin-6; TNF-α, tumor necrosis factor-alpha.

ELISA analysis showed that the model group secreted higher amounts of IL-6 and TNF-α. Empagliflozin treatment was associated with lower levels of both cytokines in the culture supernatant of AGE-BSA/high-glucose-treated C2C12 myotubes (**Figure 7**). In the additional MCP-1 ELISA experiment, AGE-BSA/high-glucose stimulation increased MCP-1 secretion, whereas empagliflozin treatment was associated with lower MCP-1 levels, particularly at 20 μM (**Supplementary Figure 3**).

## Discussion

This study evaluated the protective effect of empagliflozin in a differentiated C2C12 myotube model of inflammatory injury induced by AGE-BSA/high-glucose stimulation. The results showed that diabetic-like stimulation reduced myotube viability and enhanced AGE-RAGE/NF-κB-associated inflammatory responses, as indicated by increased RAGE expression, elevated NF-κB phosphorylation and immunofluorescence-based nuclear accumulation, and increased IL-6 and TNF-α production. Empagliflozin treatment improved cell viability and was accompanied by reduced RAGE expression, lower NF-κB phosphorylation and immunofluorescence-based nuclear accumulation, and decreased inflammatory cytokine production. These findings suggest that modulation of RAGE/NF-κB-related signaling may be involved in the anti-inflammatory effects of empagliflozin in this model; however, they do not demonstrate that this pathway is necessary or sufficient for the observed protective response.

The AGE-RAGE pathway represents an important mechanistic link between chronic hyperglycemia and tissue injury. Under diabetic conditions, persistent hyperglycemia promotes the accumulation of advanced glycation end products, which can activate RAGE and subsequently amplify downstream inflammatory signaling, including NF-κB activation. In skeletal muscle, this inflammatory microenvironment may disrupt cellular homeostasis, promote metabolic stress, and contribute to impaired muscle function. In the present study, transcriptomic enrichment analysis of diabetic skeletal muscle identified AGE-RAGE signaling and NF-κB-related inflammatory pathways, supporting the relevance of this axis to diabetes-associated skeletal muscle injury (1, 2, 5–15). Consistently, AGE-BSA/high-glucose stimulation in C2C12 myotubes reproduced key inflammatory features of this pathological process, including increased RAGE expression, elevated NF-κB phosphorylation, immunofluorescence-based nuclear accumulation of NF-κB, and increased production of IL-6 and TNF-α.

It should be noted that PI3K-Akt signaling was also enriched in the KEGG analysis and is highly relevant to skeletal muscle metabolism, insulin signaling, cell survival, and diabetes-related muscle dysfunction. Therefore, the enrichment of PI3K-Akt signaling should not be interpreted as irrelevant or excluded. Rather, it suggests that PI3K-Akt may represent another important pathway involved in the broader biological context of diabetes-related skeletal muscle injury. However, because the present experimental model was established using AGE-BSA/high-glucose stimulation and the measured endpoints mainly reflected inflammatory injury, including RAGE expression, NF-κB activation, IL-6, and TNF-α, the AGE-RAGE/NF-κB axis was considered more directly connected to the current experimental design.

Although empagliflozin is clinically used as an SGLT2 inhibitor for glycemic control, accumulating evidence suggests that its biological effects may extend beyond glucose lowering. In the present *in vitro* system, empagliflozin enhanced the survival of AGE-BSA/high-glucose-stimulated C2C12 myotubes and reduced RAGE, NF-κB, and inflammatory cytokine levels. The protective effect was most evident at 20 μM, which was therefore selected as the principal concentration for subsequent mechanistic experiments. It should also be noted that the empagliflozin concentrations used in this *in vitro* study were selected primarily based on cell viability screening and previous cell-culture experimental designs, rather than direct equivalence to clinical plasma exposure. The clinically approved oral doses of empagliflozin are 10 and 25 mg once daily, and reported peak plasma concentrations after therapeutic dosing are generally lower than the micromolar concentrations used in many *in vitro* experiments. Therefore, the concentrations used in the present study should be interpreted as pharmacological *in vitro* intervention concentrations rather than clinically equivalent free plasma concentrations. Whether the observed anti-inflammatory effects occur at clinically achievable exposure levels requires further validation using pharmacokinetic-informed concentration ranges and *in vivo* models. These findings indicate that empagliflozin may alleviate inflammatory injury in skeletal muscle cells under diabetic-like stress conditions. However, because cultured C2C12 myotubes cannot reproduce systemic glucose regulation, endocrine interactions, or the complex pathological features of diabetic sarcopenia, the current data should be interpreted as cell-based evidence of local anti-inflammatory activity rather than direct proof of therapeutic efficacy in diabetic sarcopenia (16–30).

Diabetic sarcopenia is a complex *in vivo* condition involving systemic metabolic and neuromuscular alterations, including insulin resistance, chronic low-grade inflammation, oxidative stress, mitochondrial dysfunction, altered endocrine signaling, impaired neuromuscular function, and defective muscle regeneration. Therefore, the present AGE-BSA/high-glucose-treated C2C12 myotube model should be regarded as a simplified cellular model that partially mimics hyperglycemia- and AGE-related inflammatory stress in skeletal muscle cells, rather than a complete model of diabetic sarcopenia. Accordingly, the current findings should be interpreted as preliminary *in vitro* evidence, and further validation in diabetic animal models and clinical skeletal muscle samples is required before drawing conclusions regarding the potential role of empagliflozin in diabetic sarcopenia *in vivo*.

The observed reduction in RAGE expression, NF-κB phosphorylation, and immunofluorescence-based NF-κB nuclear accumulation provides an association-level explanation for the anti-inflammatory response observed after empagliflozin treatment in this model. NF-κB nuclear translocation is a central event in inflammatory activation. In this study, immunofluorescence showed that AGE-BSA/high-glucose stimulation promoted NF-κB accumulation in the nucleus, whereas empagliflozin treatment was accompanied by reduced nuclear NF-κB accumulation. However, nuclear and cytoplasmic fractionation followed by Western blotting was not performed; therefore, these immunofluorescence findings should be interpreted as imaging-based evidence of altered NF-κB nuclear accumulation rather than biochemical confirmation of NF-κB redistribution between cytoplasmic and nuclear compartments. In parallel, qRT-PCR and ELISA demonstrated lower Il6 and Tnf mRNA expression as well as lower IL-6 and TNF-α secretion after empagliflozin treatment. Furthermore, the additional MCP-1 ELISA result provided in the **Supplementary Material** extends the inflammatory assessment from classical pro-inflammatory cytokines to chemokine-related inflammatory signaling. Because MCP-1/CCL2 is involved in inflammatory cell recruitment and has been associated with skeletal muscle inflammation and metabolic dysfunction, this supplementary result further supports the anti-inflammatory response observed after empagliflozin treatment in the present model. Taken together, these results suggest that the anti-inflammatory response to empagliflozin was accompanied by reduced activation of AGE-RAGE/NF-κB-related signaling, but causal pathway dependency remains to be verified.

It should also be emphasized that RAGE was selected as the docking target based on KEGG pathway enrichment, the AGE-BSA/high-glucose injury model, and the established role of RAGE as the receptor linking AGE stimulation to NF-κB-related inflammatory responses, rather than on PPI-based hub-gene ranking. Therefore, the docking result should not be interpreted as evidence that RAGE is the topological center of the transcriptomic network. Instead, it should be regarded as an exploratory structural hypothesis within a pathway-guided and model-driven experimental framework.

Molecular docking was performed only to explore whether a possible structural compatibility might exist between empagliflozin and RAGE. The predicted docking pose suggested that empagliflozin could be positioned within a pocket of human RAGE involving Asp160. However, this result should be interpreted strictly as a computational and hypothesis-generating observation. Docking cannot establish direct binding in living cells, target engagement, or functional regulation of the RAGE/NF-κB pathway. Moreover, the docking analysis was based on a human RAGE structure, whereas the cellular experiments were conducted in mouse C2C12 myotubes, which may introduce interspecies differences. Therefore, direct biochemical or cellular validation, such as surface plasmon resonance, isothermal titration calorimetry, cellular thermal shift assays, or other ligand–protein interaction assays, will be required to determine whether empagliflozin directly interacts with RAGE. Accordingly, the biological significance of the predicted empagliflozin-RAGE compatibility remains uncertain and should be interpreted cautiously until direct binding and functional validation experiments are performed.

Several recent studies further support the potential metabolic effects of empagliflozin in skeletal and cardiac muscle. In skeletal muscle cells, Stevanovic et al. reported that empagliflozin altered cellular energy metabolism, promoted fatty acid and leucine catabolism, decreased glucose and acetoacetate metabolism, reduced glycolysis, and was associated with AMPK activation (36). In an aging skeletal muscle model, Huang et al. showed that empagliflozin inhibited skeletal muscle fibrosis in naturally aging male mice, possibly through the AMPKα/MMP9/TGF-β1/Smad pathway (37). In cardiac muscle disease models, Rocca et al. reported that empagliflozin improved metabolic and functional outcomes in streptozotocin-diabetic and non-diabetic rats subjected to myocardial ischemia/reperfusion injury (38), while Cai et al. showed that empagliflozin improved mitochondrial dysfunction in diabetic cardiomyopathy by regulating ketone body metabolism and oxidative stress (39).

These studies indicate that empagliflozin may influence substrate utilization, glucose metabolism, fatty acid oxidation, mitochondrial function, ketone body metabolism, oxidative stress, and energy homeostasis in muscle-related contexts. Therefore, the protective response observed in AGE-BSA/high-glucose-stimulated C2C12 myotubes may not be attributable solely to reduced inflammatory signaling. It is possible that empagliflozin also improves cellular stress tolerance by modulating metabolic adaptation, mitochondrial function, or energy balance, which may secondarily influence NF-κB activation and inflammatory mediator production.

However, these cited studies were performed in different experimental settings, including human primary skeletal muscle cells, C2C12 cells, naturally aging mice, diabetic cardiomyopathy models, and myocardial ischemia/reperfusion models. Therefore, they support the biological plausibility of empagliflozin-related metabolic remodeling but do not prove that the anti-inflammatory effects observed in the present AGE-BSA/high-glucose-stimulated C2C12 myotube model are directly mediated by metabolic regulation. In the present study, metabolic endpoints such as glucose uptake, glycolytic flux, fatty acid oxidation, mitochondrial respiration, ATP production, mitochondrial membrane potential, or AMPK/Akt/PGC-1α-related signaling were not directly assessed. Therefore, the contribution of metabolic remodeling to the observed anti-inflammatory effects remains to be determined. In addition, SLC5A2 expression was not examined in C2C12 myotubes in this study; therefore, whether the observed response is SGLT2-dependent, SGLT2-independent, or related to off-target mechanisms remains unclear. Further studies are needed to clarify the relationship between empagliflozin, SGLT2 expression, and inflammatory signaling in skeletal muscle cells. Future studies should directly measure *Slc5a2* mRNA and SGLT2 protein expression in differentiated C2C12 myotubes under basal and AGE-BSA/high-glucose-stimulated conditions. In addition, SGLT2 knockdown or overexpression, together with comparative interventions using other SGLT2 inhibitors, would help determine whether the observed anti-inflammatory response is SGLT2-dependent or SGLT2-independent.

Nevertheless, the present study did not directly compare empagliflozin with other SGLT2 inhibitors, such as dapagliflozin or canagliflozin. Therefore, the observed anti-inflammatory effects should not be interpreted as being unique to empagliflozin, nor can they be concluded to represent a general class effect of SGLT2 inhibitors. Comparative studies using different SGLT2 inhibitors under the same AGE-BSA/high-glucose conditions will be required to determine whether suppression of RAGE/NF-κB-related inflammatory responses is empagliflozin-specific or shared by other members of this drug class.

### Limitations

This study has several limitations. First, all experiments were limited to an *in vitro* C2C12 myotube injury model, without validation in diabetic animal models or clinical skeletal muscle samples. Although AGE-BSA/high-glucose stimulation can partially mimic hyperglycemia- and AGE-related inflammatory stress in skeletal muscle cells, this model cannot reproduce the systemic and multifactorial pathophysiology of diabetic sarcopenia, including insulin resistance, chronic inflammation, oxidative stress, mitochondrial dysfunction, altered endocrine signaling, neuromuscular impairment, and defective muscle regeneration. Therefore, the findings cannot be directly generalized to diabetic sarcopenia or diabetic myopathy *in vivo*. In addition, the empagliflozin concentrations used *in vitro* may not directly correspond to clinically achievable free plasma concentrations, and the pharmacological relevance of these concentrations should be further examined using pharmacokinetic-informed dose ranges. Second, although the protective effect of empagliflozin was associated with changes in the RAGE/NF-κB pathway, the present study did not include RAGE gain- or loss-of-function experiments, RAGE-neutralizing antibodies, or NF-κB reverse activation assays. Therefore, we cannot definitively determine whether the anti-inflammatory effects of empagliflozin are directly dependent on RAGE/NF-κB signaling, nor can we establish whether RAGE is necessary or sufficient for the observed protective response. This issue should be considered a major limitation of the present study. In addition, NF-κB activation was evaluated by phosphorylated NF-κB protein levels and immunofluorescence-based nuclear accumulation, but nuclear and cytoplasmic fractionation followed by Western blotting was not performed. Therefore, the present study cannot biochemically confirm the redistribution of NF-κB between cytoplasmic and nuclear compartments. Future studies should quantify NF-κB p65 expression in nuclear and cytoplasmic fractions using appropriate fractionation markers, such as Lamin B1 or Histone H3 for nuclear fractions and GAPDH or β-tubulin for cytoplasmic fractions. Third, the predicted empagliflozin-RAGE interaction was derived only from molecular docking and was not validated by direct binding or target-engagement assays, such as surface plasmon resonance, cellular thermal shift assay, isothermal titration calorimetry, or other ligand–protein interaction experiments. Therefore, the docking result should not be considered definitive evidence that empagliflozin directly binds to RAGE or directly regulates the RAGE/NF-κB pathway. Fourth, the study integrated rat skeletal muscle transcriptomic data, a human RAGE structural model, and mouse C2C12 cellular experiments, which may introduce species-specific discrepancies. In addition, the present study did not establish RAGE as a topological hub within the DEG-based PPI network. Therefore, the selection of RAGE as the docking target should be regarded as pathway-guided and model-driven rather than network-topology-driven. Fifth, SLC5A2 expression was not examined in differentiated C2C12 myotubes. Therefore, the present study cannot determine whether the observed anti-inflammatory response to empagliflozin is SGLT2-dependent, SGLT2-independent, or related to off-target mechanisms. Direct assessment of *Slc5a2* mRNA and SGLT2 protein expression, together with SGLT2 knockdown or overexpression experiments, will be required in future studies. Osmotic controls were also not included, which should be addressed in subsequent experiments. In addition, although an additional native non-glycated BSA qRT-PCR control was performed and showed that native BSA did not significantly affect RAGE mRNA expression, this validation was limited to RAGE transcript levels. A complete native BSA control across downstream endpoints, including NF-κB activation, inflammatory cytokine secretion, and protein-level validation, was not performed. In addition, although PI3K-Akt signaling was enriched in the KEGG analysis, this pathway was not experimentally validated in the present study. Markers such as PI3K, phosphorylated Akt, total Akt, or downstream insulin-signaling and muscle protein metabolism-related targets were not examined. Therefore, the present study cannot compare the relative contribution of PI3K-Akt signaling with that of AGE-RAGE/NF-κB signaling. Finally, although MCP-1 was added as a supplementary chemokine marker to complement IL-6 and TNF-α, the present study still did not assess broader inflammatory, oxidative stress, metabolic, mitochondrial, muscle atrophy, or protein degradation-related markers. In particular, metabolic endpoints such as glucose uptake, glycolytic activity, fatty acid oxidation, mitochondrial respiration, ATP production, mitochondrial membrane potential, and AMPK/Akt/PGC-1α-related signaling were not examined. Therefore, the present study cannot determine whether metabolic remodeling contributes to the protective effects of empagliflozin in AGE-BSA/high-glucose-stimulated C2C12 myotubes. This limits the ability to define the broader role of empagliflozin in diabetic skeletal muscle dysfunction.

Future studies should validate the protective effects of empagliflozin in diabetic animal models, such as STZ-induced diabetic rodents or db/db mice. These studies should include functional assessments, such as grip strength, treadmill endurance, or exercise performance; histological evaluation of skeletal muscle mass, fiber cross-sectional area, and muscle fiber morphology; and molecular analyses of inflammatory cytokines, oxidative stress markers, and RAGE/NF-κB-related signaling in skeletal muscle tissue. In addition, future experiments should evaluate metabolic parameters, including 2-NBDG glucose uptake, extracellular flux analysis of mitochondrial respiration and glycolysis, fatty acid oxidation, ATP production, mitochondrial membrane potential, and AMPK/Akt/PGC-1α-related signaling, to determine whether metabolic remodeling contributes to the protective effects of empagliflozin. In addition, nuclear and cytoplasmic fractionation followed by Western blotting should be performed to further verify NF-κB p65 nuclear redistribution under AGE-BSA/high-glucose stimulation and empagliflozin treatment. To clarify whether RAGE/NF-κB signaling is required for the anti-inflammatory action of empagliflozin, RAGE siRNA/shRNA knockdown, RAGE overexpression, RAGE-neutralizing or pharmacological blockade, and NF-κB reverse activation experiments, such as TNF-α pre-stimulation, should be performed. In particular, rescue experiments under AGE-BSA/high-glucose stimulation with 20 μM empagliflozin would help determine whether restoration or activation of RAGE/NF-κB signaling weakens the protective effects of empagliflozin.

In summary, empagliflozin attenuated AGE-BSA/high-glucose-induced inflammatory injury in C2C12 myotubes. This effect was accompanied by improved cell viability, reduced RAGE expression, lower NF-κB phosphorylation and immunofluorescence-based nuclear accumulation, and decreased IL-6 and TNF-α production. These cell-based findings suggest that modulation of RAGE/NF-κB-related inflammatory signaling may be involved in the protective effects of empagliflozin. However, additional RAGE gain- or loss-of-function experiments, NF-κB rescue assays, and animal or clinical validation are required before a causal pathway dependency or therapeutic relevance can be established.

## Data Availability

The original contributions presented in the study are included in the article/**Supplementary Material**. Further inquiries can be directed to the corresponding authors.
