# Supplementary material for: Empagliflozin attenuates AGE-BSA/high-glucose-induced inflammation in C2C12 myotubes associated with suppression of the RAGE/NF-κB pathway
Source: Front Endocrinol (Lausanne). 2026 Aug 18;17:1903580. doi: 10.3389/fendo.2026.1903580 (PMC13529554; doi:10.3389/fendo.2026.1903580)
Supplement: Supplementary file 1 [file SupplementaryFile1.docx]

**Supplementary Figure S1. Validation of C2C12 myotube differentiation.**

Representative bright-field and immunofluorescence images showing C2C12 cells after 5 days of differentiation in medium containing 2% horse serum. Differentiated cells displayed elongated and fused myotube-like morphology under light microscopy. Myosin heavy chain staining further confirmed the formation of differentiated myotubes. Nuclei were counterstained with DAPI. Scale bar = 20 μm.

**Supplementary Figure S2. Native non-glycated BSA did not affect RAGE mRNA expression in C2C12 myotubes.**

Differentiated C2C12 myotubes were treated with native non-glycated BSA (Servicebio, Wuhan, China; Cat. No. GC305006) at the same concentration and duration as AGE-BSA treatment, namely 200 μg/mL for 48 h. RAGE mRNA expression was then detected by qPCR. Native non-glycated BSA did not significantly alter RAGE mRNA expression compared with the blank control group, suggesting that BSA itself did not markedly induce RAGE expression under the present experimental conditions. The primers used for qPCR were the same as those described in the main Methods section. Data are presented as mean ± SD; n = 4 independent biological replicates. ns, not significant.

**Supplementary Figure S3. Empagliflozin reduced MCP-1 secretion in AGE-BSA/high-glucose-stimulated C2C12 myotubes.**

Differentiated C2C12 myotubes were exposed to AGE-BSA (200 μg/mL) and high glucose (30 mmol/L) and treated with empagliflozin at the indicated concentrations for 48 h. MCP-1 concentrations in the culture supernatants were measured using ELISA. Data are presented as mean ± SD; n = 4 independent biological replicates per group. ****P < 0.0001 versus the blank control group; #P < 0.05 and ###P < 0.001 versus the AGE-BSA/high-glucose group; ns, not significant.
